# The Design of Alapropoginine, a Novel Conjugated Ultrashort Antimicrobial Peptide with Potent Synergistic Antimicrobial Activity in Combination with Conventional Antibiotics

**DOI:** 10.3390/antibiotics10060712

**Published:** 2021-06-13

**Authors:** Ali Salama, Ammar Almaaytah, Rula M. Darwish

**Affiliations:** 1 Department of Pharmaceutics and Pharmaceutical Technology, School of Pharmacy, University of Jordan, Amman 11942, Jordan; asalama@meu.edu.jo; 2 Department of Pharmacy, Faculty of Pharmacy, Middle East University, Amman 11831, Jordan; 3 Department of Pharmaceutical Technology, Faculty of Pharmacy, Jordan University of Science and Technology, Irbid 22110, Jordan

**Keywords:** antimicrobial peptides, synergistic, antibiotics, rational design

## Abstract

(1) Background: Antimicrobial resistance represents an urgent health dilemma facing the global human population. The development of novel antimicrobial agents is needed to face the rising number of resistant bacteria. Ultrashort antimicrobial peptides (USAMPs) are considered promising antimicrobial agents that meet the required criteria of novel antimicrobial drug development. (2) Methods: Alapropoginine was rationally designed by incorporating arginine (R), biphenylalanine (B), and naproxen to create an ultrashort hexapeptide. The antimicrobial activity of alapropoginine was evaluated against different strains of bacteria. The hemolytic activity of alapropoginine was also investigated against human erythrocytes. Finally, synergistic studies with antibiotics were performed using the checkerboard technique and the determination of the fractional inhibitory index. (3) Results: Alapropoginine displayed potent antimicrobial activities against reference and multi-drug-resistant bacteria with MIC values of as low as 28.6 µg/mL against methicillin-resistant S. aureus. Alapropoginine caused negligible toxicity toward human red blood cells. Moreover, the synergistic studies showed improved activities for the combined conventional antibiotics with a huge reduction in their antimicrobial concentrations. (4) Conclusions: The present study indicates that alapropoginine exhibits promising antimicrobial activity against reference and resistant strains of bacteria with negligible hemolytic activity. Additionally, the peptide displays synergistic or additive effects when combined with several antibiotics.

## 1. Introduction

Antimicrobial resistance (AMR) and the emergence of multi-drug-resistant (MDR) bacteria remains one of the most serious challenges being faced by world health authorities worldwide [1]. Recent decades have witnessed an upsurge in the number of MDR bacteria with some being reported to display panresistance against all clinically available antimicrobials, ushering the possibility of humans entering the postantibiotic era and consequently threatening the lives of millions of people around the globe [2]. Several regional and international health organizations have been consistently reporting the emergence of pan-drug-resistant (PDR) Gram-negative bacteria displaying resistance against the most powerful antimicrobial agents still available in the clinic such as colistin [3,4,5]. This situation is exacerbated by the recent COVID-19 pandemic which swept all over the globe and shifted the focus of all health authorities, governments, and pharmaceutical companies on developing vaccines and antiviral medications to tackle the COVID-19 pandemic and consequently overshadowing the issue of AMR. This situation is only worsened by the fact that the pharmaceutical industry has made little contribution to the development of antimicrobials in recent decades and its pipelines remain dry with only fewer classes of antimicrobial agents managing to reach the clinic [6,7]. Accordingly, there is a significant need to develop new classes of antimicrobial agents capable of eradicating the escalating number of MDR and PDR strains of bacteria.

Antimicrobial peptides (AMPs) represent a promising class of potential antimicrobial agents due to their potency, efficacy, and broad-spectrum activity against various strains of microorganisms [8,9]. These peptides may provide alternative substitutes to traditional antibiotics which are currently facing multiple resistance from various strains of bacteria [10,11].

AMPs are positively charged amphipathic molecules and constitute one of the major pillars of the natural innate host defense system of a large number of living organisms [12]. AMPs display a positive charge ranging from (+3–+9) and exhibit 30–50% hydrophobicity in general [13]. These physicochemical properties play a major role in AMPs proposed mechanism of antimicrobial activity as their positive charge is responsible for the electrostatically driven interaction with the negatively charged membranes of bacteria allowing AMPs with their amphipathic structures to form pores in target membranes which consequently leads to intracellular leakage followed by cell death [14]. AMPs act as the initial line of defense against a large number of microorganisms such as viruses, bacteria, protozoa, and fungi [15].

Despite the numerous efforts to advance AMPs into clinical use and their several advantages mentioned previously, several obstacles have limited their development and entry into the clinic. These obstacles are mainly related to AMPs innate poor stability in vivo due to the activity of serum proteases in addition to their high manufacturing costs and poor target selectivity, a factor that renders these molecules toxic when advanced in clinical trials [16].

Recent research efforts have focused on the development of alternative platforms for AMPs design and development that are aimed at reducing AMPs toxicity, manufacturing costs, and enhancement of in vivo stability. These efforts include the design of hybrid peptides, sequence modification, and the use of unnatural D-amino acids to enhance stability and reduce the toxicity of AMPs [17].

In this study, we aim to target the obstacles challenging AMP development through the design of conjugated ultrashort antimicrobial peptides (USAMPs). The short sequence of USAMPs is expected to reduce the toxicity and manufacturing costs of the peptides [18], while the conjugated part will act as an anchor that will provide the hydrophobic portion needed for the peptide’s bacterial membrane interaction and permeability. The designed USAMP was challenged not only with reference strains of bacteria but also with MRSA and ESBL *E. coli* which are considered problematic bacteria, especially in hospital-acquired infections. Moreover, the study aims at exploring the synergistic activity of combining the USAMP with conventional antibiotics and consequently the impact of peptide-antibiotic synergism on reducing the effective antimicrobial concentrations of both the peptide and the antibiotic. Accordingly, we report the synthesis and antimicrobial activity of alapropoginine, a novel conjugated ultrashort antimicrobial peptide with potent antimicrobial activities against reference and clinical isolates of multi-drug-resistant bacteria. These effective antimicrobial concentrations were associated with negligible toxicity toward human red blood cells. Moreover, the synergistic studies showed improved activities for the combined conventional antibiotics with a huge reduction in their minimum inhibitory concentration values. The concentration of alapropoginine was reduced by 75% in some of the peptide–antibiotic combinations.

## 2. Results

### 2.1. Alapropoginine Design, Synthesis, and Purification

**Figure 1:**
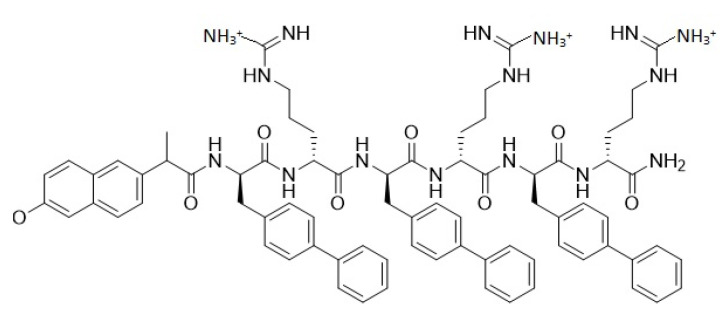
The chemical structure of alapropoginine at physiological pH.

Alapropoginine, a hexa ultrashort conjugated antimicrobial peptide was designed rationally through the use of alternating subunits of both arginine (R) and biphenylalanine (B). The design strategy depended on choosing positively charged amino acids as representatives of the cationicity needed for electrostatic interactions with the negatively charged bacterial membranes. Antimicrobial peptides require a delicate balance between cationicity and hydrophobicity to bind effectively to bacterial membranes and elicit their membrane disrupting activities. Accordingly, biphenylalanine was chosen to represent the hydrophobic moieties needed to balance the hexapeptide. Additionally, the hexapeptide was conjugated to 2-(6-methoxynaphthalen-2-yl)propanoic acid to maintain and enhance the antibacterial activity of the conjugated molecule as several studies reported that 2-(6-methoxynaphthalen-2-yl)propanoic acid conjugation was beneficial in increasing the antibacterial potency of several agents [19]. Alapropoginine displays a net positive charge of +3 and a molecular weight of 1367.64 Daltons, Figure 1 represents the overall structure of Alapropoginine. The hexapeptide was purified to >98% purity using RP-HPLC (Supplementary Figure S1) and its identity was confirmed by electrospray ionization mass spectrometry (ESI-MS) with the synthetic peptide displaying major peaks in the +2 and +3 charge state of 684.6 and 456.8 Daltons (Figure S2). The detailed synthetic process of alapropoginene is described in detail within the supplementary material.

### 2.2. In Vitro Antimicrobial Activity of Alapropoginine

Alapropoginine’s antimicrobial activity was evaluated against different reference and resistant strains of Gram-positive and Gram-negative bacteria. The bacterial strains employed in this study included reference Gram-positive strains of *S. aureus* (ATCC 29215) and methicillin-resistant *S. aureus* (MRSA) (ATCC BAA-41). For Gram-negative bacteria, a reference strain of *E. coli* (ATCC 25922) in addition to ESBL *E. coli* (ATCC BAA-3054) was employed to determine the MIC and MBC of alapropoginine.

As shown in Table 1, alapropoginine displayed potent activity against Gram-positive bacterial strains with a minimum inhibitory concentration (MIC) of 9.152 µg/mL against the reference strain *S. aureus* and MIC of 17.16 µg/mL against MRSA (ATCC BAA-41). As for the Gram-negative bacteria, the MIC value reported for the reference strain of *E. coli* was 20.5 µg/mL and 28.6 µg/mL against ESBL *E. coli*. The MBC values were the same as the MIC values against the four bacterial types indicating that the peptide is behaving in a bactericidal manner.

### 2.3. Hemolytic Activity of Alapropoginine

The ability of alapropoginine to damage mammalian erythrocytes, in particular, was assessed using the standard erythrocytes hemolysis assay. The erythrocytes were challenged with different concentrations of alapropoginine ranging from 5.72–114.4 µg/mL. The obtained results revealed that the alapropoginine caused only 1% hemolysis after 60 min of incubation with human erythrocytes at a concentration of 114.4 µg/mL (Table 2). The hemolytic assay clearly indicates that the peptide exhibits negligible hemolytic activity.

### 2.4. Determination of the MIC and MBC of the Individual Antibiotics

The eight different antibiotics employed in this study were challenged with control and multidrug-resistant strains of Gram-positive and Gram-negative bacteria to determine the antimicrobial activity of each antibiotic against the employed bacterial strains. All the reported data of MIC and MBC values of antibiotics are summarized in (Table 3). As reported, the MIC values of levofloxacin, rifampicin, cefixime, and amoxicillin were equal to the MBC values. This is an indication of the bactericidal behavior of these antibiotics. On the contrary, the bacteriostatic behavior is reported for chloramphenicol, clarithromycin, vancomycin, and doxycycline due to higher MBC values needed to kill the bacterial strains when compared to the MIC values.

### 2.5. Synergistic Activity of Alapropoginine in Combination with Conventional Antibiotics

The antibacterial activity of *Alapropoginine* in combination with eight conventional antibiotics (levofloxacin, chloramphenicol, rifampicin, amoxicillin, clarithromycin, vancomycin, cefixime, and doxycycline) was assessed using the conventional broth microdilution method [20,21]. Alapropoginine was combined with the eight conventional antibiotics using the checkerboard technique to determine the synergistic response of these combinations on the antimicrobial potency of both alapropoginine and the individual antibiotics, respectively. The increase in the antibacterial potency as a result of synergism is reported by calculating the FIC indices which indicate if the peptide antibiotic combination was synergistic (FIC ≤ 0.5), additive (FIC 0.5 < FIC ≤ 1), indifferent (1 < FIC ≤ 4), or antagonist (FIC > 4) in effect [22].

Alapropoginine in combination with eight antibiotics was tested against the four bacterial strains involved in this study. As summarized in (Table 4), a significant drop in MIC values was reported for several peptide-antibiotic combinations. Out of the 32 combinations, six combinations exhibited a synergistic effect against reference and resistant strains of Gram-positive and Gram-negative bacteria. Moreover, 14 combinations displayed an additive effect. The last 12 combinations only displayed an indifferent effect. Regarding the methicillin-resistant strain MRSA (ATCC BAA-41), the *alapropoginine*–vancomycin combination displayed a synergistic effect with an FIC index of 0.5. For the clinically isolated resistant strain of EBSL *E. coli*, only the *alapropoginine*–chloramphenicol combination exhibited a synergistic effect with an FIC index of 0.38.

The synergistic effect of *alapropoginine*–vancomycin combination led to a decrease in the MIC value of *alapropoginine* by 73.3% when compared with its individual MIC. The MIC value of Vancomycin decreased by 75% compared to the individual MIC of the antibiotic. For the control strain of *S. aureus*, the *alapropoginine*–levofloxacin and *alapropoginine*–vancomycin combinations displayed synergistic effects with 0.12 and 0.3 FIC values, respectively. The MIC values of alapropoginine decreased by 90% with levofloxacin and by 75% with vancomycin. Additionally, the synergism led to a drop in the MIC values of both levofloxacin and vancomycin by 99.8% and 95%, respectively. For the reference control strain of the Gram-negative Strain of *E. coli*, both combinations of the *alapropoginine*–levofloxacin and *alapropoginine*–rifampicin displayed a synergistic effect with FIC index values equal to 0.26 and 0.37, respectively. In these two combinations, the MIC of alapropoginine decreased by 86% when combined with levofloxacin and 66.7% with rifampicin. Furthermore, the MIC of both antibiotics levofloxacin and rifampicin decreased by 87.5% and 96.7%, respectively. For the Gram-negative ESBL *E. coli* clinically isolated strain, only the *alapropoginine*–chloramphenicol combination managed to produce a synergistic effect with FIC indices equal to 0.37. Interestingly, amoxicillin was not active against ESBL *E. coli* individually. However, when combined with alapropoginine, an additive effect was observed that resulted in the enhancement of the antimicrobial activity of amoxicillin and a reported FIC index of 1. The other combinations displayed additive or indifferent effects with FIC values in the range of (0.5 < FIC ≤ 1) or (1 < FIC ≤ 4), respectively.

## 3. Discussion

Antimicrobial resistance represents one of the major global health threats to the human population [23]. Some even consider the issue as a neglected global crisis requiring immediate international intervention [24]. The problem of AMR could be further escalated currently by the emergence of the COVID-19 pandemic and its implications on the policies adopted by governments and research institutions which could lead to allocating substantial funds for antiviral research rather than the development of novel antimicrobials which could only worsen the AMR dilemma [25]. Antibiotics which were and still are considered as the major backbone of clinical medicine are becoming ineffective against various strains of Gram-positive and Gram-negative bacteria [26]. The development of new antimicrobial agents has slowed down in recent decades with only a few antibiotics that mainly target Gram-positive bacteria entering the clinic [27]. This situation necessitates an urgent call for public, governmental and private institutions to accelerate and support antimicrobial development efforts to avoid a future catastrophic event where the emergence and spread of panresistant bacteria could become inevitable [28].

Antimicrobial peptides have received great attention as potential candidates for antimicrobial drug development due to their broad-spectrum antimicrobial activity [29]. Recently and due to difficulties and challenges facing AMPs development into clinically active agents as a result of their toxic nature and high manufacturing costs, recent efforts have focused on the development of ultrashort antimicrobial peptides (USAMPs). This group of peptides exhibit several advantages such as short amino acid sequences which would facilitate large-scale production of these peptides on an economical scale [30]. Additionally, USAMPs in general display decreased hemolytic activity when compared with their longer counterparts. The design of USAMPs has to be carefully articulated to create a delicate balance between the structural motifs and features that allow AMPs to attach to bacterial membranes and cause cell lysis [31]. AMPs cause cell lysis by relying on two major physicochemical property-related events that are crucial for peptide antimicrobial activity. The first event is related to the cationic nature of the peptide as the positively charged amino acids interact with the negatively charged phospholipids of bacterial membranes, this electrostatic interaction between the peptides and the bacterial membranes allows AMPs to aggregate in large numbers on the surface of bacterial membranes and facilitating the initiation of the second event which mainly depends on the hydrophobic nature of the peptide and allows AMPs to orient themselves properly with the amphipathic bacterial membrane layer and penetrate the hydrophobic lipid tail and consequently cause cell lysis and death [32]. In our study, we have relied on these general assumptions in the design of alapropoginine which is an ultrashort six-amino-acid AMP that displays a net cationic charge of +3. It is believed that the optimal positive charge required for proper and sufficient electrostatic interaction between AMPs and bacterial membranes is in the range of (+3–+6). Alapropoginine is composed of three arginine amino acids which are responsible for the +3 net positive charge of the peptide in addition to three unnatural amino acids represented by biphenylalanine that was specifically chosen to be included in the peptide design as several reports and structural simulation studies are reporting that biphenylalanine acts as a membrane anchor in USAMPs and contributes heavily in the hydrophobic membrane penetration needed for bacterial membrane’s pore formation. Alapropoginine was also conjugated to a carbon side chain composed of 2-(6-methoxynaphthalen-2-yl)propanoic acid. The rationale behind the conjugation strategy is to sustain the hydrophobic grip of the biphenylalanine amino acids and provide a hydrophobic stabilizer to the peptide [33].

As displayed in our study, the design strategy proved to be successful in generating a conjugated USAMP with potent antimicrobial activity against reference strains and clinical isolates of Gram-positive and Gram-negative bacteria. The reported MIC for alapropoginine against clinically isolated ESBL *E. coli* was 28.6 µg/mL while the MIC against MRSA was 17.16 µg/mL, respectively. Interestingly, the hemolytic activity of alapropoginine against mammalian RBCs was nearly not detected even at concentrations reaching 114.4 µg/mL, an indicator that the peptide displays minimal hemolytic activity. This data does not elucidate the full cytotoxic potential of the peptide and should be further explored in future toxicity studies. The minimal hemolytic activity could be explained by the inability of USAMPs to adsorb efficiently on the membranes of RBCs as the electrostatic interactions between the peptide and the mammalian membranes are weaker when compared with their bacterial counterparts due to the zwitterionic nature of mammalian membranes [34]. Additionally, a proposed explanation for the inability of USAMPs to damage RBCs could be attributed to the abundant presence of cholesterol moieties in mammalian membranes that could enhance the rigidity of the membranes and consequently challenge USAMPs ability to bend and penetrate these membranes [35].

Several studies have reported that combining AMPs with antibiotics will provide enhanced microbiocidal activity for both the peptide and the antibiotic. However, the exact mechanism of action responsible for the additive or synergistic effect of combining both AMPs and antibiotics is still unclear. It has been suggested the combination strategy may provide a cooperative mode of cell membrane permeabilization through a self-promoted uptake mechanism [36]. However, the major hypothesis regarding the synergistic mode of action remains linked to AMPs ability to destabilize bacterial membranes and thus enhance the accessibility of the antibiotics to their intracellular targets which would ultimately lead to an additive or synergistic effect [37].

Our results display that of the 32 combinations employed in the study, six combinations exhibited a synergistic effect against reference and resistant strains of Gram-positive and Gram-negative bacteria. Moreover, 14 combinations displayed an additive effect. For MRSA, the *alapropoginine*–vancomycin combination produced a synergistic effect with an FIC index of 0.5, while the *alapropoginine*–chloramphenicol combination produced a significant synergistic effect against ESBL *E. Coli*. The mechanism responsible for the synergistic effects of the peptide–antibiotics combinations is unclear yet, one hypothesis for the synergistic effect proposes that the destruction and pore formation effects of USAMPs in bacterial membranes enhance the intracellular entry and thus facilitating their target delivery and the accomplishment of their antimicrobial function rapidly [38]. However, the main limitations of our study are related to the inability to provide a full cytotoxicity profile of alapropoginine and this issue has to be further elucidated in future studies. Alapropoginine’s cytotoxicity should be evaluated against mammalian cells in vitro in addition to in vivo studies to generate evidence regarding the success of the conjugation strategy in reducing AMPs toxicity. Additionally, the synergistic mode of action should be further investigated in mechanistic studies capable of identifying the rationale behind the peptide antibiotic synergy.

The peptide–antibiotic combination has proved to be a successful strategy in several previously studied peptides and could provide a feasible option for the development of AMPs as the synergistic effect could lead to a decrease in the effective concentrations of AMPs to nanomolar concentrations [39].

## 4. Materials and Methods

### 4.1. Design and Synthesis of Alapropoginine

Alapropoginine was rationally designed by incorporating alternating subunits of both arginine (R) and biphenylalanine (B) to create an ultrashort hexapeptide. The peptide was further conjugated with 2-(6-Methoxy-2-naphthyl) propionic acid. Alapropoginine was synthesized using conventional solid-phase Fmoc chemistry (GL Biochem, Shanghai, China). Alapropoginine was synthesized following standard Fmoc solid-phase protocols on Wang resin. Peptide elongation was effected using standard HBTU coupling chemistry in dimethylformamide (DMF) solvent with a fourfold molar excess of diisopropyl ethylamine (DIEA) in *N*-methyl-2-pyrrolidone (NMP) and a threefold molar excess of each Fmoc-protected amino acid or 2-(6-methoxynaphthalen-2-yl) propanoic acid. Alapropoginine was cleaved from the resin, using 95% trifluoroacetic acid (TFA), 2.5% triisopropylsilane, and 2.5% thioanisole (3 h, room temperature), and precipitated using cold (−20 °C) diethyl ether. The synthesized peptide’s purity was determined by reverse-phase high-performance liquid chromatography (RP-HPLC). The identification of alapropoginine was confirmed by mass analysis and through the employment of electrospray ionization mass spectrometry (ESI-MS).

### 4.2. Determination of the Minimum Inhibitory Concentrations (MICs) and Minimum Bactericidal Concentrations (MBCs) for Alapropoginine

Using sterile 96-well polypropylene microtiter plates, the microbroth dilution method as outlined by the Clinical and Laboratory Standards Institute (CLSI) guidelines was adopted to determine the MIC and MBC of alapropoginine. In brief, Muller–Hinton broth (MHB) was used as the growth medium for the different bacterial strains following their removal from frozen glycerol. Bacterial cells were grown overnight in MHB and diluted to 10^6^ CFU/mL in the same medium before use. Different dilutions of Alapropoginine were prepared accordingly. In separate 96-well microtiter plates, 50 μL of each peptide and 50 µL of diluted bacterial suspension were added to each well. Each plate included six replicates of each peptide concentration divided into six wells. The plate was incubated for 18 h at 37 °C. The growth of bacteria was determined by measuring OD at *λ* = 570 nm by an ELISA plate reader. MIC was determined accordingly (as the lowest concentration of antimicrobial drugs which is needed to inhibit the growth of the bacteria). Each plate included a positive control column which was composed of 50 μL of bacterial suspension plus 50 μL MHB without any antimicrobial agents and a negative control column composed of 100 μL of MHB to ensure bacterial growth and the sterility of MHB, respectively.

MBC was determined by taking 10 μL from the clear negative wells, and turbid positive control wells and they were streaked on sterile labeled nutrient media agar and incubated for 24 h at 37 °C. The lowest concentration that led to having <0.1% viable cells (killing 99.9%) was referred to as the MBC value. The experiments were performed in triplicate.

### 4.3. MIC and MBC Determination of Individual Antibiotics

MICs and MBCs were determined against reference bacterial strains of *S. aureus*, *E. coli,* and resistant clinical isolates of extended-spectrum beta-lactamase (ESBL) *E. coli* and methicillin-resistant *S. aureus* (MRSA) via preparing different concentrations of each antibiotic. All antibiotic solutions were dissolved in water then diluted in the sterile broth. MICs and MBCs determination were performed in triplicate.

### 4.4. MIC Determination of Alapropoginine in Combination with Antibiotics

According to the broth microdilution checkerboard technique [40], MICs of peptide-antibiotic combinations against reference bacterial strains of *S. aureus*, *E. coli*, ESBL *E. coli,* and MRSA were tested and determined as described in Section 2.3. However, in this assay, each microtiter well contained a mixture of alapropoginine and one antibiotic at different concentrations, at a volume of 25 µL of alapropoginine concentration and 25 µL of each antibiotic concentration. These combinations were added to six wells of a sterile flat–bottomed 96 well-plate that contained 50 µL of the diluted bacterial suspension. MICs determination was performed in triplicate.

### 4.5. Determination of the Synergistic Activity of Alapropoginine

The fractional inhibitory concentration (FIC) is the summation of the inhibitory concentration values of each antimicrobial component in the antimicrobial combination divided by the inhibitory concentration of the individual antimicrobial agent [41].

The FIC indices were interpreted as follows: ≤0.5: synergistic activity, 0.5–1: additive activity, 1–4 indifferent, >4: antagonistic. Interpretation and assessment of the FIC index and antimicrobial activity of peptides-antibiotics combinations were conducted according to the broth microdilution checkerboard technique [22,23].

### 4.6. Erythrocyte Hemolytic Assay

The Erythrocyte Hemolytic assay was performed to determine the ability of the Alapropoginine to cause hemolysis to human erythrocytes. Two ml of human blood (Zen-bio Inc., Durham, NC, USA) was placed into a 50 mL centrifuge tube, centrifuged at 3000× *g* for 5 min. The supernatant was discarded and the cell pellet was suspended in 48 mL of PBS and centrifuged at 3000× *g* for 5 min; this step was repeated three times. Finally, the cell pellet was resuspended in a sterile tube containing 50 mL PBS to reach a final concentration of 4% RBCs. This was followed by the addition of 1 mL of each peptide concentration to 1 mL of erythrocyte suspension. Controls were prepared by the addition of 5 µL of Triton X-100 to 1 mL of RBC suspension (positive control). The blank (negative control) was prepared by adding 1 mL of RBC suspension to 1 mL of phosphate-buffered saline (PBS). The suspension was incubated for 60 min at 37 °C. Tubes were gently vortexed and 1 mL of each sample was aspirated and placed into sterilized Eppendorf tubes and then centrifuged for 5 min at 3000× *g*. From each supernatant 100 µL was placed into a 96-well plate. Absorbance was measured at λ = 570 nm with the aid of a microplate reader. The hemolysis percentage was calculated according to the following equation [24]:

$$\%\;\mathrm{Hemolysis}=\frac{\left(A\;-\;\mathrm{AO}\right)\;}{\left(\mathrm{AX}\;-\mathrm{AO}\right)}\times 100$$

where A is OD 450 with the peptide solution,

A0 is OD 450 of the blank.
And A is OD 450 of control (0.1% Triton X-100).

## 5. Conclusions

In conclusion, we report, the design and antimicrobial characterization of a novel conjugated ultrashort antimicrobial peptide with potent activities against clinically resistant isolates of Gram-positive and Gram-negative bacteria and negligible hemolytic activities. The peptide displayed several synergistic activities when combined when conventional antibiotics and could prove to be a significant candidate for further antimicrobial development.

## Figures and Tables

**Table 1:** MIC and MBC values of alapropoginine against the four bacterial strains employed in the study.

**Gram-Positive Strains**	**ATCC**	**MIC Value (µg/mL)**	**MBC Value (µg/mL)**
*S. aureus*	29215	9.152	9.152
MRSA	BAA-41	17.16	17.16
**Gram-Negative Strains**	**ATCC**	**MIC Value (µg/mL)**	**MBC Value (µg/mL)**
*E. coli*	25922	20.5	20.5
ESBL *E. coli*	BAA-3054	28.6	28.6

**Table 2:** Hemolytic activity of *alapropoginine* against human erythrocytes after 60 minutes’ incubation. The results were recorded at λ = 450 nm.

Concentration (µg/mL)	Hemolysis %
5.72	0
11.44	0
22.88	0
45.76	0
68.64	0
91.52	0
114.4	1

**Table 3:** The minimum inhibitory and minimum bactericidal concentrations (µg/mL) of the eight antibiotics against the tested bacterial strains.

Antibiotics	*S. aureus* (ATCC 29215)	MRSA *(ATCC* BAA-41)	*E. coli* (ATCC 25922)	ESBL *E. coli* (BAA-3054)
	MIC-(MBC)	MIC-(MBC)	MIC-(MBC)	MIC-(MBC)
Levofloxacin	0.57-(0.57)	11.44-(11.44)	2.28-(2.28)	13.7-(13.7)
Chloramphenicol	22.8-(34.32)	28.6-(45.7)	91.5-(114.4)	171-(228.8)
Rifampicin	0.028-(0.028)	0.0057-(0.0057)	17.6-(17.65)	57-(57)
Amoxicillin	5.72-(5.72)	45.7-(45.7)	28.6-(28.6)	228.8-(286)
Clarithromycin	0.57-(1.71)	143-(171)	143-(171)	143-(228.8)
Doxycycline	2.2-(11.44)	11.44-(22.88)	1.7-(17.6)	18.3-(28.6)
Vancomycin	5.72-(5.72)	2.28-(2.28)	228.8-(228.8)	286-(286)
Cefixime	4.57-(4.57)	34.32-(34.32)	6.8-(6.8)	91.5-(91.5)

**Table 4:** Combinatorial antimicrobial activity of alapropoginine and antibiotics including the fractional inhibitory concentrations (FIC) indices for the antimicrobial combinations against tested bacterial species. MICs are expressed in µg/mL.

Bacterial Strains	Antibiotic	Antibiotic MIC	Antibiotic/Alapropoginine MICs	*Alapropoginine* MIC	*Alapropoginine/Antibiotic* MIC	
*S. aureus* (ATCC 29215)	Levofloxacin	0.57	0.057	9.152	0.143	0.12
	Chloramphenicol	34.3	11.4	9.152	2.2	0.58
	Rifampicin	0.025	0.015	9.152	0.57	0.66
	Amoxicillin	5.7	2.8	9.152	6.86	1.25
	Clarithromycin	1.7	0.57	9.152	4.5	0.83
	Doxycycline	11.4	2.2	9.152	4.5	0.7
	Vancomycin	0.5	0.028	9.152	2.2	0.3
	Cefixime	0.57	1.144	9.152	4.5	0.75
MRSA *(ATCC* BAA-41)	Levofloxacin	11.4	9.152	17.1	6.86	1.2
	Chloramphenicol	45.7	22.8	17.1	6.86	0.9
	Rifampicin	0.005	0.0025	17.1	11.4	1.17
	Amoxicillin	45.7	28.6	17.1	11.4	1.3
	Clarithromycin	228.8	91.5	17.1	17.1	1.4
	Doxycycline	22.8	11.4	17.1	6.86	0.9
	Vancomycin	2.2	0.57	17.1	4.5	0.5
	Cefixime	34.3	17.1	17.1	9.152	1.03
*E. coli* (ATCC 25922)	Levofloxacin	2.2	0.28	20.5	2.8	0.26
	Chloramphenicol	114.4	28.6	20.5	11.4	0.81
	Rifampicin	17.1	0.57	20.5	6.8	0.37
	Amoxicillin	28.6	9.15	20.5	9.15	0.76
	Clarithromycin	171	114.4	20.5	13.7	1.33
	Doxycycline	17.1	6.8	20.5	9.15	0.84
	Vancomycin	171	114.4	20.5	11.4	1.22
	Cefixime	6.8	2.2	20.5	4.57	0.56
ESBL *E. coli* (BAA-3054)	Levofloxacin	13.7	11.4	28.6	17.1	1.43
	Chloramphenicol	228.8	17.1	28.6	8.58	0.38
	Rifampicin	68	6.8	28.6	17.1	1.1
	Amoxicillin	250	171.6	28.6	11.4	1
	Clarithromycin	228.8	143	28.6	17.1	1.23
	Doxycycline	28.6	11.4	28.6	11.4	0.8
	Vancomycin	228.8	171.6	28.6	17.1	1.35
	Cefixime	91.5	22.8	28.6	17.1	0.85

## Data Availability

The data are available upon request by email form the authors.

## Supplementary Materials

The following are available online at https://www.mdpi.com/article/10.3390/antibiotics10060712/s1, Figure S1: Analytical RP-HPLC chromatogram of the Alapropoginine, Figure S2: Positive electrospray ionization (ESI) mass spectrometric (MS) analysis of Alapropoginine.
