# Supplementary material for: The Design of Alapropoginine, a Novel Conjugated Ultrashort Antimicrobial Peptide with Potent Synergistic Antimicrobial Activity in Combination with Conventional Antibiotics
Source: Antibiotics (Basel). 2021 Jun 13;10(6):712. doi: 10.3390/antibiotics10060712 (PMC8231522; doi:10.3390/antibiotics10060712)
Supplement: Supplementary file 1 [file antibiotics-10-00712-s001.zip › antibiotics-1257996-supplementary.pdf]

antibiotics

MDPI

Supplementary Material

The Design of Alapropoginine, a Novel Conjugated Ultrashort Antimicrobial Peptide with Potent Synergistic Antimicrobial Activity in Combination with Conventional Antibiotics

Ali Salama [1,2], Ammar Almaaytah [2,3,*] and Rula M. Darwish [1,*]

Figure S1. Analytical RP-HPLC chromatogram of the Alapropoginine. Alapropoginine was purified using a gradient HPLC system, fitted with an analytical column (Inertsil ODS-SP; 0.46 cm × 25 cm), and was eluted with a linear gradient (41%-100%) of (v/v) TFA/acetonitrile and 0.1 (v/v) TFA/water over 30 min, at a flow rate of 1 ml/min.

Sample Description:
Structure:BR-6
Number: 0200046
Lot No:P191125-CL764852
Column: 4.6mm*250mm, Inertsil ODS-SP
Solvent A: 0.1%Trifluoroacetic in 100% Acetonitrile
Solvent B: 0.1%Trifluoroacetic in 100% Water

Gradient:

	A	B
0.01min	41%	59%
25.00min	66%	34%
25.01min	100%	0%
30.00min	Stop	

Flow rate:1.0ml/min
Wavelength:220nm
Volume:10 μ l

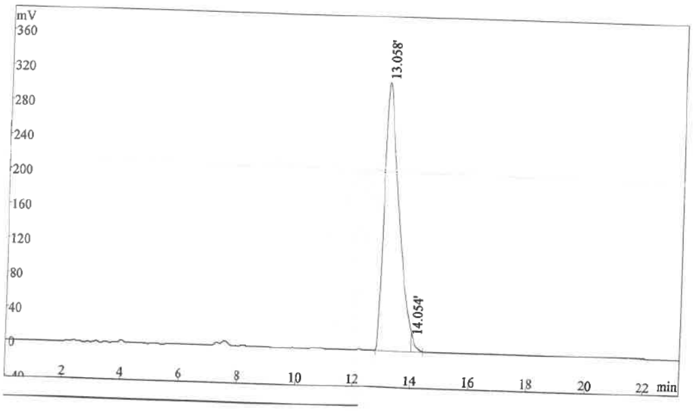

Rank	Time	Conc.	Area	Height
1	13.058	98.78	10697101	309337
2	14.054	1.219	131956	15257
Total		100	10829057	324594

Figure S2. Positive electrospray ionization (ESI) mass spectrometric (MS) analysis of Alapropoginine showing major peaks in +2 and +3 charge state of 684.6 and 456.8 Daltons.

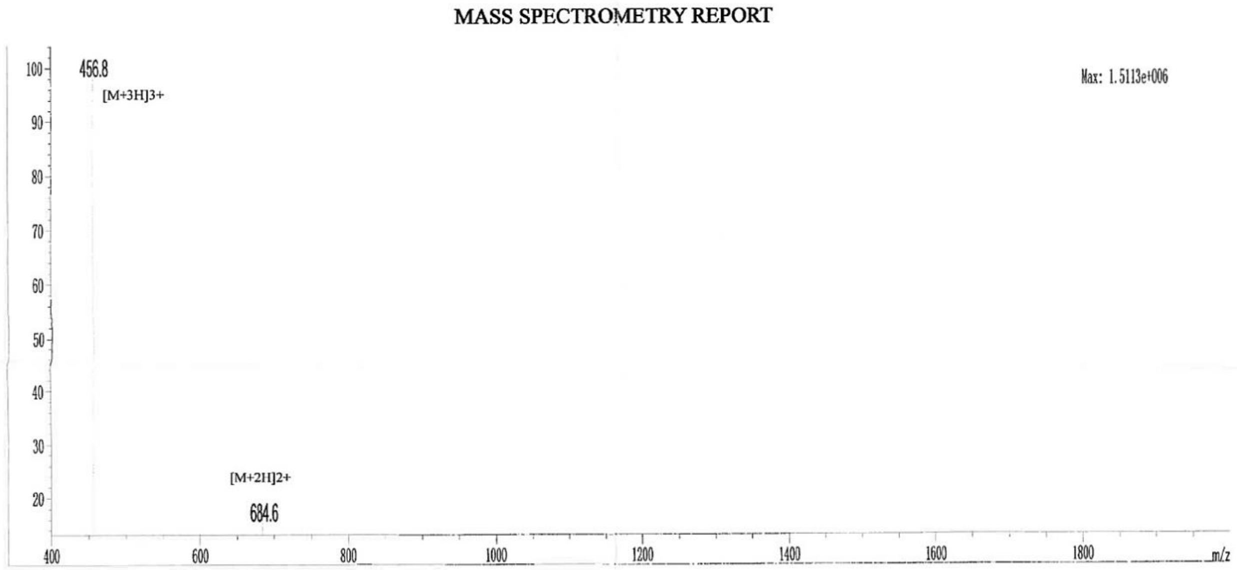

Sample Description		Instrument	Agilent-6125B		
Analyzed date:	2019-12-09	Probe:	ESI	Probe Bias:	+4.5kv
Analyst:	YU	Nebulizer Gas Flow:	1.5L/min	Detector:	1.5kv
Sample:	BR-6	CDL:	-20.0v	T. Flow:	0.2ml/min
M.W.:	1367.64	CDL Temp.:	250 °C	B. Conc.:	50%H2O/50%ACN
Lot. No.:	P191125-CL764852	Block Temp.:	200 °C		

Synthesis of Alapropoginine

Alapropoginine was synthesized following standard Fmoc solid phase protocols on Wang resin. Peptide elongation was effected using standard HBTU coupling chemistry in dimethylformamide (DMF) solvent with fourfold molar excess of diisopropyl ethylamine (DIEA) in N-methyl-2-pyrrolidone (NMP) and a threefold molar excess of each Fmoc-protected amino acid or 2-(6-methoxynaphthalen-2-yl) propanoic acid. Alapropoginine was cleaved from the resin, using 95% trifluoroacetic acid (TFA), 2.5% triisopropylsilane and 2.5% thioanisole (3 h, room temperature), and precipitated using cold (−20 °C) diethyl ether.
